# CELLULAR RESPIRATION AND RESILIENCE AS A POTENTIAL BIOLOGICAL MECHANISM DRIVING SEX DIFFERENCES IN AGING

**DOI:** 10.1093/geroni/igac059.1735

**Published:** 2022-12-20

**Authors:** Adam Salmon, Daniel Adekunbi

**Affiliations:** The University of Texas Health Science Center at San Antonio, San Antonio, Texas, United States; University of Wyoming, Laramie, Wyoming, United States

## Abstract

There are substantial differences in the progression of aging between males and females including in progression and prevalence of disease and longevity. Not all can be explained solely by sex-specific endocrine regulation and growing evidence suggests there are basic biological and genetic differences in sex that drive disparity in physiological function. In this study, we describe metabolic differences at the cellular level that both define some of these biological differences as well as provide a potential mechanism for delineating relevant molecular mechanisms of aging. Using HET3 mice, a genetically heterogeneous model with a consistent female advantage in longevity, we show that primary fibroblast lines retain functional metabolic characteristics, including mitochondrial response and stress resilience, which are representative of the sex of the donor animal. These differences persist even after several rounds of passage using standard culturing techniques suggesting these differences are not driven by direct impact of circulating sex hormones. Moreover, we find that differences in these cellular responses have some predictive power to determine both sex- and individual-specific responses to physiological challenge including obesity and longevity. In addition, we are able to use this model to delineate how donor sex affects cellular responses within defined pillars of aging including proteostasis, metabolic function, and adaption to stress. Overall, our model then provides valuable in defining the cellular responses that contribute to the mammalian aging process.

